# Using Digital Media to Improve Adolescent Resilience and Prevent Mental Health Problems: Protocol for a Scoping Review

**DOI:** 10.2196/58681

**Published:** 2024-10-16

**Authors:** Riris D Rachmayanti, Fatwa Sari Tetra Dewi, Diana Setiyawati, Hario Megatsari, Rian Diana, Retno Vinarti

**Affiliations:** 1 Department of Epidemiology, Biostatistic, Population Study and Health Promotion Public Health Faculty Universitas Airlangga Surabaya Indonesia; 2 Doctoral Program in Public Health Faculty of Medicine Public Health and Nursing Universitas Gadjah Mada Yogyakarta Indonesia; 3 Department of Health Behavior, Environment and Social Medicine Faculty of Medicine, Public Health and Nursing Universitas Gadjah Mada Yogyakarta Indonesia; 4 Center for Public Mental Health Faculty of Psychology Universitas Gadjah Mada Yogyakarta Indonesia; 5 Innovation in Health Communication, Information, and Education Research Group Universitas Airlangga Surabaya Indonesia; 6 Department of Information Systems Faculty of Intelligent Electrical and Information Technology Institut Teknologi Sepuluh Nopember Surabaya Indonesia

**Keywords:** adolescents, digital media, mental health, resilience, scoping review

## Abstract

**Background:**

Global databases show a high prevalence of mental health problems among adolescents (13.5% among those aged 10-14 years and 14.65% for those aged 15-19 years). Successful coping depends on risk and protective factors and how their interaction influences resilience. Higher resilience has been shown to correlate with fewer mental health problems. Digital mental health interventions may help address these problems.

**Objective:**

This protocol serves as a framework for planning a scoping review to map the types of digital communication media and their effectiveness in increasing resilience in youths.

**Methods:**

The Joanna Briggs Institute guidelines will be used: defining the research questions; identifying relevant studies; study selection (we will select articles based on titles and abstracts); charting the data; collating, summarizing, and reporting the results; and consultation. The synthesis will focus on the type of digital media used to increase adolescent resilience skills and the impact they have on adolescent resilience skills. Quantitative and qualitative analyses will be conducted.

**Results:**

The study selection based on keywords was completed in December 2023, the study screening and review were completed in February 2024, and the results manuscript is currently being prepared. This scoping review protocol was funded by the Center for Higher Education Funding and the Indonesia Endowment Fund for Education.

**Conclusions:**

The results of the study will provide a comprehensive overview of commonly used digital media types and their effectiveness in increasing youth resilience. Thus, the results of this scoping review protocol can serve as foundational evidence in deciding further research or interventions. This study may also be used as a guideline for mapping and identifying the type and impact of communication media used to increase adolescents’ resilience skills.

**International Registered Report Identifier (IRRID):**

DERR1-10.2196/58681

## Introduction

### Background

Positive mental health is the desired outcome of health promotion interventions [1]. Preventive and promotive mental health interventions have been shown to be feasible and effective in many age groups and settings [2]. Promoting adolescent mental health is the most effective and proactive technique to prevent mental illness and disability now and in the future [3]. Mental health problems with varying symptoms are characterized by abnormal thoughts, perceptions, emotions, behaviors, and relationships with others [4].

Adolescents are vulnerable to mental health problems due to puberty, body, and hormonal changes; changes in their social environment; and changes in the brain and mind. Mental illnesses such as depression, anxiety, eating disorders, substance use disorders, and psychosis appear for the first time before the age of 24 years [5]. Global databases show that the prevalence of adolescent mental health problems is as much as 13.5% for those aged 10-14 years and 14.65% for those aged 15-19 years [6]. Adolescent mental health problems emerge due to many factors, including social determinants such as parent or family condition, peer group, socioeconomic status, and community and local services [7].

Being able to deal with problems depends on the associated risks and protections. The interaction between risk and protection affects resilience [8]. Resilience is an individual’s ability to function in adversity or stress. Adolescents with high resilience can face life difficulties more easily as they become adults [9], and high adolescent resilience has been shown to be related to fewer mental health problems [10]. Resilience refers to successfully adapting to stress and an individual’s ability to maintain or regain homeostasis despite experiencing adverse life events [11].

The use of media to build adolescents’ health literacy is one method used to increase youth resilience, and activities online can contribute to youths’ resilience. Thus, social work interventions can harness online media to help promote resilience [12]. Digital interventions have been shown to improve resilience [13,14], maintain emotional regulation [15,16], and help users self-monitor [17]. Adolescents can build resilience by practicing making safe decisions when faced with risks, helping them construct supportive connections [18]. Additionally, adolescents who are more resilient offline are also more resilient online [12]. Bad mental health events may have an impact on the future of children and teenagers younger than 18 years. The mental health of children and adolescents is the foundation for their mental health as adults [10].

Digital technology has been linked to many positive outcomes related to information, peer support, professional online support, and more informal benefits, such as distraction and social connection [12]. Digital mental health interventions electronically deliver information, support, and therapy to treat, alleviate, or manage symptoms of mental health conditions [19]. Digital mental health interventions have been proven to be beneficial for addressing mental health problems [20].

With digital interventions, technological barriers, cost-effectiveness, and technical challenges exist, including fragmented and unsustainable systems, lack of clear standards, unreliable available data, and infrastructure and capacity gaps [21]. Challenges specific to digital health interventions include user involvement, user suitability, and the accuracy of the digital intervention [22]. Despite these challenges, media can still contribute to the treatment and prevention of mental health problems, including by helping build resilience to improve adolescents’ abilities to prevent mental health issues. This systematic review will address the following:

- Map the impact of digital communication media on increasing adolescents’ resilience
- Map the types and features of digital communication media that increase resilience in the adolescent population

This protocol will use evidence-based methods for the mapping of the types of digital mental health interventions.

### Objectives

A protocol describes details, provides a clear understanding of the authors’ research questions, and ensures transparency and reproducibility. Protocols allow systematic reviewers to plan carefully and thereby anticipate potential problems; allow reviewers to explicitly document what is planned before they start their review, enabling others to compare the protocol and the completed review, replicate review methods if desired, and judge the validity of the planned methods; and prevent arbitrary decision-making about study selection and data extraction [23]. A protocol for a scoping review makes the research objectives, methods, reporting, and process more transparent. Protocols help researchers set clear goals for reflection, predict possible obstacles and anticipate them, evaluate the success of the process and the results of the review, and prevent duplication of other research [24]. Protocols provide transparency to the process, help manage the review, minimize bias (a risk of bias assessment will not be performed in this scoping review), and list references to digital health interventions for other researchers to review. This protocol aims to guide and manage a scoping review (research objectives, methods, study selection, extraction, and reporting) for identifying the types, effectiveness, and benefits of digital communication media used to intervene in youth resilience.

## Methods

### Overview

This review will follow the Joanna Briggs Institute (JBI) guidelines for scoping reviews [25,26]. Additionally, Arksey and O’Malley [27] have outlined the following six steps for scoping reviews: (1) defining the research questions; (2) identifying relevant studies; (3) study selection; (4) charting the data; (5) collating, summarizing, and reporting the results; and (6) consultation. The PRISMA-ScR (Preferred Reporting Items for Systematic Reviews and Meta-Analyses Extension for Scoping Reviews) checklist will also be used. Searching the literature, refining the search strategy, assessing articles for inclusion, and collecting pertinent data will be done iteratively.

### Protocol Registration and Report Information

A PRISMA checklist is provided in Multimedia Appendix 1. The checklist is an effective tool for guiding the reporting of scoping studies [28,29]. This protocol was registered on the Open Science Framework (3CNRT) [30] on July 7, 2023.

### Research Questions

The following research questions were proposed:

1. What are the characteristics of digital mental health interventions being carried out to prevent adolescent mental health problems?
2. What type of content is being used for digital mental health interventions to prevent adolescent mental health problems?
3. Does digital communication media affect the resilience abilities of adolescents and reduce mental health problems?

### Eligibility Criteria

The research questions previously mentioned and the eligibility criteria were developed using the Population, Intervention, Comparator, Outcomes (PICO) framework (Multimedia Appendix 2). Researchers use PICO to identify the key concepts of a scoping review, develop appropriate search terms to describe the problem, and determine inclusion and exclusion criteria.

#### Population

The population in this study are adolescents (also known as youth, young people, teenagers); adolescent, based on the World Health Organization definition, refers to the phase of life between childhood and adulthood, from ages 10 to 19 years [31]. Sawyer et al [32] described adolescents as people aged 10-24 years. This review will include all studies reporting on adolescents aged 10-24 years who are registered as students.

#### Intervention

This review will include interventions that use communication media for digital health. Digital health refers to the development and adoption of modern information and communication technology systems to help various health system stakeholders interact and make informed decisions about their health, the health of others, and the health system [33]. Digital interventions for health promotion or risk prevention can be distributed through mobile devices (eg, smartphones and tablets), computer- and web-based programs, social media apps, telemonitoring devices (eg, external sensors or smartwatches), and games [34]. Digital media includes the internet, social media, and mobile tools, while nondigital media includes television, print, radio, etc [35]. In this scoping review, the articles can include all intervention-related digital communication or digital media (websites, mobile apps, chat rooms, the internet, social media, and other digital media).

#### Comparator

Comparators or comparisons refer to groups that were compared to the intervention group [36]. The comparison can be to another treatment or intervention or no treatment at all [37]. In this scoping review, articles will be included if the comparator or comparison does not use digital or nondigital media (eg, television, radio, or printed media such as leaflets, posters, and brochures).

#### Outcomes

Outcome refers to the measure of interest or the study’s target end points that are objectively defined. There may be only primary outcome measures or additional and secondary outcome measures [38]. In this scoping review, the articles must include the outcome of resilience (coping mechanism, coping stress, positive mental health) and well-being on mental health (stress, anxiety, depression, eating disorder, bipolar disorder, posttraumatic stress disorder, schizophrenia, and suicide).

### Identifying Relevant Studies

Stage 2 of the review includes two steps. The first step is to identify all scoping reviews that are relevant and were published by August 2023. The search was conducted on the Cochrane and Scopus databases with an unlimited time period. A total of 6 scoping reviews were found related to digital mental health and adolescents [39-44]. However, these 6 reviews were more focused on anxiety in adults, implementing digital health interventions for people experiencing homelessness or youths who dropped out of school, and developing digital health interventions for native aboriginal people. No scoping review focused on the effectiveness of digital mental health for increasing resilience in adolescents. The second step is to identify randomized controlled trials on digital mental health interventions used to boost resilience and prevent adolescent mental health problems.

### Search Strategy

Studies will be identified by conducting a literature review, establishing a search strategy, and searching for peer-reviewed articles and gray literature. The screening will start with the title, followed by the abstract and full text of selected studies. Studies published between 2000 and 2023 will be included. The search strategy and inclusion and exclusion criteria have been established and approved.

A search for relevant studies will be done in a structured manner using four databases: ProQuest, Scopus, Cochrane Library (CENTRAL), and *JMIR Mental Health*. Gray literature will be searched for through GreyNet International. We will also hand search for articles by selecting appropriate references from the articles eligible for analysis and other relevant publications. All search results and the screening process will be exported to Rayyan’s free online systematic review management software. Articles that match the inclusion criteria will be included (Multimedia Appendix 3). The authors will use Medical Subject Headings (MeSH) to search databases using keywords related to mental health, resilience, youth, digital, communication, and media (Multimedia Appendix 4). The PRESS (Peer Review of Electronic Search Strategies) guideline developed in 2015 has six steps: translation; Boolean and proximity operators; subject headings; text word searching; spelling, syntax, and line number; and limits and filters [45]. In addition, the PICO elements will be used as a guide for screening and identifying studies.

### Study Selection

The study search results obtained in stage 2 will be exported to Rayyan for data management (removing duplicates and referencing) [46,47]. The screening of titles, abstracts, and full texts will be conducted by three independent reviewers based on the inclusion and exclusion criteria. Studies that meet the inclusion criteria will be included in the full-text screening. Otherwise, they will be excluded, with the reasons for exclusion presented in the final report. Three independent reviewers will conduct the data extraction. The final results will be presented in the form of a PRISMA diagram [28]. Disagreements or discrepancies that arise will be resolved by discussion or consensus.

### Charting (Extracting) the Data

#### Data Extraction

This study used three reviewers for data extraction to determine and discuss eligible articles. Reviewers will sift through the same data and discuss the results of the screening of the titles, abstracts, and full texts; if there is easy access to the full text; and the key PICO elements. The reviewer team will resolve any disagreements by using the judgment of the third reviewer. Three experienced verifiers will verify the search results. An Excel (Microsoft Corporation) sheet will be created to extract essential information from the selected studies (Multimedia Appendix 5).

#### Data Synthesis

The synthesis will follow the JBI guidelines. This synthesis focuses on the type of media used to intervene in adolescent resilience skills and the impact the media had on adolescent resilience skills. The quantitative analysis will be presented descriptively with frequency distribution tables and statistical summaries of the article characteristics. The synthesis will use evidence source details and characteristics (citation details, country, context, and participants). The synthesis will also detail the results extracted from the sources (in relation to the scoping review’s concept) concerning the type of media and its impact. Researchers will read the selected articles and develop themes based on the main results. Themes will be created by determining the type of digital media used (game, website/internet based, mobile app, or computer based) and the media’s effectiveness or impact (effective or noneffective), determined by *P* values <.05 or <.001 depending on the statistical method used in the eligible manuscript. Themes will be developed while analyzing the results of the eligible articles. Two other researchers will validate the results of the thematic analysis. If there are differences, they will be discussed and resolved together. Each article will be categorized based on the research location, methodology, and participant demographics.

### Collating, Summarizing, and Reporting the Results

The eligible references from the scoping review will be exported into Mendeley (the list of databases will be exported in RIS format with full-text references). A PRISMA flowchart will be used to visualize the sequence of the literature screening process for eligible articles for analysis. A literature map will then be used to match and categorize the relevant references. Later, a descriptive analysis and tabulation will be used to make a narrative summarization.

### Consultation

Discussion between consultants and the research team will review and refine preliminary findings, aiming to reach a consensus. A psychiatrist will be consulted when additional data or information is needed. Follow-up discussions will be done for further clarifications. Additionally, consultations will be done to improve the results’ accuracy and provide additional information and meaning [28].

### Ethical Considerations

There will be no involvement of humans, animals, patients, or the public in this review. This review will synthesize data from peer-reviewed studies that have been published. Thus, no ethical approval was required.

## Results

The selection of studies based on keywords was completed in December 2023. Meanwhile, study screening and study review were completed in February 2024, and preparation of the scoping review’s results manuscript is currently underway. The scoping review is expected to be completed by the end of November 2024. This paper will describe the following findings: research selection, research characteristics (percentages based on the total eligible manuscripts), description of digital health, synthesis of results (type of media, content of media, targeting accessibility, and the effectiveness or the effect and impact of the intervention related to adolescent mental health), and evaluation, including strengths, limitations, and recommendations for research. This scoping review is supported by the Ministry of Education as funding for a scholarship program (Indonesian Educator Scholarship), which was awarded for 2022-2025. Funders were not involved in the development of the protocol.

## Discussion

Previous studies have shown that technology can help improve the resilience capabilities of youths aged 10-19 years [12]. Digital technologies can impact multiple areas of development for adolescents in an increasingly digital world, including engendering positive outcomes in terms of the brain, cognition, social-emotional development, and mental health [48]. The role of digital health in mitigating risk and increasing resilience has been extensively studied [13]. Digitalization has impacted the ability of supply chains to mitigate disruption and build resilience [49]. Digital technology was also shown to enhance resilience during the COVID-19 pandemic [50]. During the pandemic, five things were found to strengthen resilience when using digital technology, namely, distributed training, local expertise, regional autonomy and ownership, local infrastructure, and platform design infrastructure including in the form of digital information [51]. Digital platforms can also increase individual resilience for overcoming mental health problems [52].

This protocol acts as an effective preplan, providing clarity to the study’s flow and considering how to communicate the outcome data to the reader [53]. All scoping reviews must begin with a well-defined topic and a carefully developed and detailed protocol, guiding the conduct and reporting of the review while providing transparency and minimizing reporting bias [54]. Protocols are highly recommended before beginning a scoping review [55].

Some challenges may arise in this study. First, few articles might be found on the topic, considering that research in digital mental health and resilience is still not widely studied. To mitigate this, we have added a registered database, gray literature, and hand searching to our review. Second, there may be too few randomized controlled trials on digital health interventions to increase resilience. To mitigate this, we will include all types of experiments. Third, if the initial screening finds a vast number of articles, the keywords may be too broad. To mitigate this, we will use specific keywords. Fourth, studies on mental health outcomes related to chronic illness, situations during the pandemic, and other disease problems outside of mental health may emerge. Therefore, keywords need to be specified, and the initial screening stage (abstract selection) needs to be stringent [56].

The results of the study will provide a complete picture of the types of digital media that are used for increasing youth resilience and their effectiveness. Media has been shown to influence adolescent resilience abilities [57], so the integration of digital media in interventions may help decrease adolescent mental health problems [58]. Thus, the results of this scoping review can be used as a basis for evidence in determining further research or interventions. This scoping review only focuses on digital communication media.

Our dissemination plan includes presentations at conferences and for relevant stakeholders, and publication in a peer-reviewed journal. One limitation of this study is that improving resilience skills is influenced by other factors such as the environment, social support, social skills, and socioeconomic status. Including these variables would allow for further analysis.

This scoping review will provide a comprehensive overview of the existing literature on the use of communication media for improving resilience skills in adolescents.

## Appendix

Multimedia Appendix 1 PRISMA (Preferred Reporting Items for Systematic Reviews and Meta-Analyses) checklist.

Multimedia Appendix 2 Population, Intervention, Comparator, Outcomes (PICO) framework.

Multimedia Appendix 3 Inclusion and exclusion criteria.

Multimedia Appendix 4 Searching strategy.

Multimedia Appendix 5 Data extraction variables.

## Abbreviations

JBI: Joanna Briggs Institute
MeSH: Medical Subject Headings
PICO: Population, Intervention, Comparator, Outcomes
PRESS: Peer Review of Electronic Search Strategies
PRISMA-ScR: Preferred Reporting Items for Systematic Reviews and Meta-Analyses
