# Correction: Using Digital Media to Improve Adolescent Resilience and Prevent Mental Health Problems: Protocol for a Scoping Review

**DOI:** 10.2196/67759

**Published:** 2024-12-05

**Authors:** Riris D Rachmayanti, Fatwa Sari Tetra Dewi, Diana Setiyawati, Hario Magatsari, Rian Diana, Retno Vinarti

**Affiliations:** 1 Department of Epidemiology, Biostatistic, Population Study and Health Promotion Public Health Faculty Universitas Airlangga Surabaya Indonesia; 2 Doctoral Program in Public Health Faculty of Medicine Public Health and Nursing Universitas Gadjah Mada Yogyakarta Indonesia; 3 Department of Health Behavior, Environment and Social Medicine Faculty of Medicine, Public Health and Nursing Universitas Gadjah Mada Yogyakarta Indonesia; 4 Center for Public Mental Health Faculty of Psychology Universitas Gadjah Mada Yogyakarta Indonesia; 5 Innovation in Health Communication, Information, and Education Research Group Universitas Airlangga Surabaya Indonesia; 6 Department of Information Systems Faculty of Intelligent Electrical and Information Technology Institut Teknologi Sepuluh Nopember Surabaya Indonesia

In “Using Digital Media to Improve Adolescent Resilience and Prevent Mental Health Problems: Protocol for a Scoping Review” (JMIR Res Protoc 2024;13:e58681) the authors made one addition.

The following affiliation was added for the first author:

Doctoral Program in Public Health, Faculty of Medicine Public Health and Nursing, Universitas Gadjah Mada, Yogyakarta, Indonesia

This will be labelled as "affiliation 2", with all the subsequent affiliations being renumbered accordingly.

The correction will appear in the online version of the paper on the JMIR Publications website on December 5, 2024, together with the publication of this correction notice. Because this was made after submission to PubMed, PubMed Central, and other full-text repositories, the corrected article has also been resubmitted to those repositories.

