# Supplementary material for: Using Digital Media to Improve Adolescent Resilience and Prevent Mental Health Problems: Protocol for a Scoping Review
Source: JMIR Res Protoc. 2024 Oct 16;13:e58681. doi: 10.2196/58681 (PMC11525077; doi:10.2196/58681)
Supplement: Multimedia Appendix 2 [file resprot_v13i1e58681_app2.pdf]

Multimedia Appendix 2 PICO Framework

Participants/Population	The population in this study is teenagers from various age groups<br>Youth<br>Young adults<br>Adolescent<br>Teenagers
Intervention(s)/Exposure(s)	Digital Communication media, namely all media used and from various sources relevant to mental health problems. Can be:<br>Web-based<br>Application<br>Chat rooms<br>Interactive media<br>Film<br>Blogs<br>Digital media<br>Internet<br>Social media
Comparator(s)/Control(s)	Does not use media or no digital media or non-digital
Outcomes	Resilience<br>Mental health (stress, anxiety, depression, eating disorder, bipolar disorder, post-traumatic stress disorder, schizophrenia, and suicide)
