# Supplementary material for: Using Digital Media to Improve Adolescent Resilience and Prevent Mental Health Problems: Protocol for a Scoping Review
Source: JMIR Res Protoc. 2024 Oct 16;13:e58681. doi: 10.2196/58681 (PMC11525077; doi:10.2196/58681)
Supplement: Multimedia Appendix 3 [file resprot_v13i1e58681_app3.pdf]

Multimedia Appendix 4 Inclusion and Exclusion Criteria

Inclusion Criteria	Exclusion Criteria
• At least 50% of study participants are identified as adolescents, young adults, youth, and teenagers.<br>• A minimum of 50% of the study are aged 10-24.<br>• Study design randomized control trial.<br>• Media used for intervention are websites, blogs, mobile applications, interactive media, chat rooms, digital media, internet-based, or other digital devices.<br>• The study focuses on mental health or well-being outcomes, including stress, anxiety, depression, eating disorder, bipolar disorder, post-traumatic stress disorder, schizophrenia, and suicide.<br>• Language: English.	• It is unrelated to mental health or well-being (such as physical health and nutritional status).<br>• Adult Population.<br>• Study design of cohort, qualitative study, observational study, literature review, systematic review, and meta-analysis.<br>• The study focuses on developing media, user-centered design, user experience testing, and developing instruments.<br>• Language: Non-English.
