# Supplementary material for: Using Digital Media to Improve Adolescent Resilience and Prevent Mental Health Problems: Protocol for a Scoping Review
Source: JMIR Res Protoc. 2024 Oct 16;13:e58681. doi: 10.2196/58681 (PMC11525077; doi:10.2196/58681)
Supplement: Multimedia Appendix 4 [file resprot_v13i1e58681_app4.pdf]

Multimedia Appendix 2: Search Strategy

Search Terms (Mesh Term)

Selected databases: Scopus, Proquest, Cochrane Library (CENTRAL), JMIR Mental Health, JMIR mHealth and uHealth, Journal of Medical Internet Research

Search terms
MeSH (((("Adaptation, Psychological"[Mesh]) OR "Mental Health"[Mesh]) OR "Adolescent Health"[Mesh]) AND "Communications Media"[Mesh])

Published date: All Years

Resource types: Articles
