# Supplementary material for: Using Digital Media to Improve Adolescent Resilience and Prevent Mental Health Problems: Protocol for a Scoping Review
Source: JMIR Res Protoc. 2024 Oct 16;13:e58681. doi: 10.2196/58681 (PMC11525077; doi:10.2196/58681)
Supplement: Multimedia Appendix 5 [file resprot_v13i1e58681_app5.pdf]

Multimedia Appendix 5 Data Extraction Variables.

Variable	Description
Study characteristics	Author, date/years, funder, title, journal, volume, issue, pages, country, objectives of the study, and study design
Description of digital health	Purpose, type of digital health/technology, target population, length (words), duration (mins), owner
Variables relating to the process	
Participant demographics	Country, age, gender, role (school/university students, patients)
Methodology used	RCT (duration of intervention and implementation), sample size, data collection (community or school)
Variable relating to research	
Type of media	• Mobile application, web pages, digital videos, software, internet intervention, and related digital media<br>• Reasoning for why using that platform
Content of Media	• Information/ Messages content<br>• Treatment, Prevention<br>• Features such as (chatbot, video, screening, diagnostic, direct chat, etc.)
Targeting accessibility	To whom and how the medium was used
Effectiveness (The effect/impact of the platform related to adolescent mental health)	Feasibility and Effectiveness trial for participants or platforms related to adolescent mental health (increasing or decreasing of adolescent mental health with P value) .
Evaluation process (author reflections)	Reported results, strengths, limitations, and recommendations
